# An open source software for fast grid-based data-mining in spatial epidemiology (FGBASE)

**DOI:** 10.1186/1476-072X-13-46

**Published:** 2014-10-30

**Authors:** David M Baker, Alain-Jacques Valleron

**Affiliations:** Institut National de la Santé et de la Recherche Médicale (U986), Bicêtre Hospital, Paris-Sud University, Paris, France; Université Pierre et Marie Curie, Paris, France

**Keywords:** Computational epidemiology, Cluster, Environmental factors, Software, Geographical grid, Type 1 diabetes

## Abstract

**Background:**

Examining whether disease cases are clustered in space is an important part of epidemiological research. Another important part of spatial epidemiology is testing whether patients suffering from a disease are more, or less, exposed to environmental factors of interest than adequately defined controls. Both approaches involve determining the number of cases and controls (or population at risk) in specific zones. For cluster searches, this often must be done for millions of different zones. Doing this by calculating distances can lead to very lengthy computations. In this work we discuss the computational advantages of geographical grid-based methods, and introduce an open source software (FGBASE) which we have created for this purpose.

**Methods:**

Geographical grids based on the Lambert Azimuthal Equal Area projection are well suited for spatial epidemiology because they preserve area: each cell of the grid has the same area. We describe how data is projected onto such a grid, as well as grid-based algorithms for spatial epidemiological data-mining. The software program (FGBASE), that we have developed, implements these grid-based methods.

**Results:**

The grid based algorithms perform extremely fast. This is particularly the case for cluster searches. When applied to a cohort of French Type 1 Diabetes (T1D) patients, as an example, the grid based algorithms detected potential clusters in a few seconds on a modern laptop. This compares very favorably to an equivalent cluster search using distance calculations instead of a grid, which took over 4 hours on the same computer. In the case study we discovered 4 potential clusters of T1D cases near the cities of Le Havre, Dunkerque, Toulouse and Nantes. One example of environmental analysis with our software was to study whether a significant association could be found between distance to vineyards with heavy pesticide. None was found. In both examples, the software facilitates the rapid testing of hypotheses.

**Conclusions:**

Grid-based algorithms for mining spatial epidemiological data provide advantages in terms of computational complexity thus improving the speed of computations. We believe that these methods and this software tool (FGBASE) will lower the computational barriers to entry for those performing epidemiological research.

**Electronic supplementary material:**

The online version of this article (doi:10.1186/1476-072X-13-46) contains supplementary material, which is available to authorized users.

## Background

Examining whether disease cases are clustered in space is an important part of epidemiological research (see [1–6]). Another important part of spatial epidemiology is testing whether patients suffering from a disease are more, or less, exposed to some environmental factors of interest (see [7–11]). Cluster search fits into a hypothesis-free approach, while testing the effect of specific environmental factors fits into a hypothesis-driven approach. Both these approaches are important: the hypothesis-driven approach generally provides higher statistical power since fewer statistical tests are performed, whereas the hypothesis-free approach can discover associations which researchers did not think of. Searching for clusters consists simply in locating regions where the number of cases (relative to controls or relative to the population at risk) is larger than would be expected by chance. This is a data-driven approach in the sense that the researcher does not formulate any a priori hypothesis regarding the data. Instead the data is examined to see if it exhibits any patterns, which may not have been immediately visible. If such patterns are discovered, they may suggest further specific explorations, and help identify unexpected environmental risk factors.

Testing the effect of specific environmental factors requires the researcher to specify environmental entities (factories, waste dumps, freeways, etc.). The disease impact of the exposition to these spatial entities is often quantified using odds ratios, and when possible, dose-response effects which may suggest a causal relationship. Because the researcher must formulate an a priori hypothesis, namely the existence of link between a set of spatial entities and the disease, this fits into a hypothesis-driven approach. After the hypothesis is chosen, the data is examined to see whether or not it supports the hypothesis.

In practice, both approaches benefit from the popularization of geographic information systems. Addresses of patients with a disease of interest are routinely geolocalized, dozens of databases inform on air and water quality, soil cover, climate, local social environment, etc.. with an always increasing spatial resolution. Using exact locations (as opposed to grouping by administrative units, as in the past) comes at a computational cost, which can be quite large particularly for cluster searches. A popular method for performing a cluster search involves moving a shape (called a scanning window) across the region of study and counting the number of cases and controls in the scanning window. A common method used for example by the software SaTScan™ [12, 13] is to use circular (or elliptical) scanning windows. After each move, the number of cases and the number of controls (or the population at risk) enclosed in the scanning window is evaluated and a statistic is computed. The statistic is used to evaluate whether the number of cases in the scanning window is large enough to be unlikely due to chance. The window is generally circular, so every time it moves, the distance between its center and each case/control must be calculated. In order to yield good results, the scanning window should take many different positions on the map. In addition, several different sized scanning windows should be used in order to detect small as well as large clusters. The statistic derived by Kulldorff in [1] is well suited for this purpose. Kulldorff derives a statistical likelihood ratio test for cluster zones based on a binomial model. Let p be the probability of an individual inside the zone Z being a case, and q the probability of an individual outside this zone Z being a case. Let *n*_*z*_ be the number of individuals inside the zone Z and let *c*_*z*_ be the number of cases inside the zone Z. Finally let C be the total number of cases in the region of study and N the total number of individual in the region of study. The null hypothesis is that the zone Z is not a cluster (i.e. *H*_0_ : *p* = *q*) and the alternative hypothesis is that the zone Z is a cluster (i.e. *H*_1_ : *p* > *q*). The likelihood ratio is:


As the denominator of the likelihood ratio is the same for all zones, only the numerator matters when looking for the most likely clusters.

Kulldorff shows that if  then *sup*_*p* >*q*_*L*(*z*, *p*, *q*) equal to:


And if  then *sup*_*p* >*q*_*L*(*z*, *p*, *q*) is equal to *p*^*c*^(1 - *p*)^*N* - *C*^.

Circular scanning windows and distance calculations works well when cases are localized to a small number of administrative units. However, when each case and each control is localized to its exact location, the computational time involved can become very large. Consider the following example, which gives an order of magnitude of the amount of calculations involved. When collection of scanning windows scans the map of France with 1km steps, a fixed size window will take over half a million positions. Multiply that by six to allow for different sized windows. Recall that after each move the distance from the scanning window to every case/control must be computed. For a population study of 10,000 individuals, this amounts to *thirty billion distance calculations*. The computational burden is compounded by the fact that these are not simply Euclidian distances, but distances on a sphere which are more computationally intensive.

**Figure 1:**
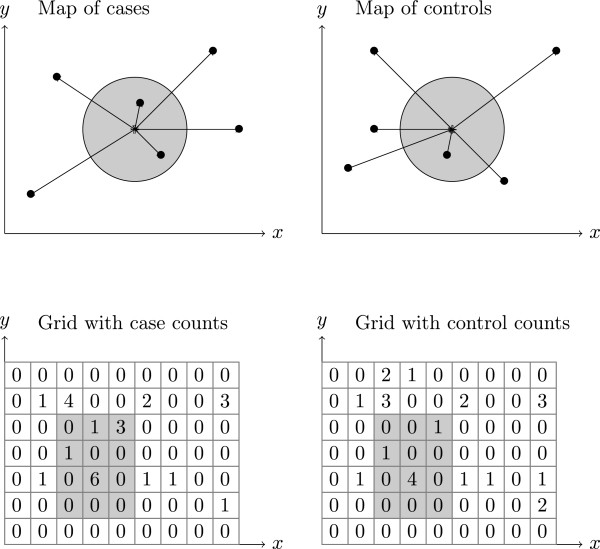
**Comparison of the use of point locations with distance computations versus the use of the grid-based approach when searching for clusters.** (Above): Setting where cases and controls data consists of points (identified by latitude and longitude). Determining the number of cases and controls in a (circular) region requires computing the distances from the center of the region to each case and each control. For cluster searches, millions of regions (the potential clusters) are considered which means billions of distance calculations for a medium size (case/control) study. (Below): Setting where cases and controls have been projected to a grid. Determining the number of cases and controls in a (rectangular) region requires only reading values from memory-mapped arrays and summing them. This method is very fast even for cluster searches where millions of regions are considered (the potential clusters).

The initial motivation of this work was to downsize this computational issue by developing a grid approach that we found requires considerably less computational time (see Figure 1). The goal of this paper is to introduce the corresponding open source software that we have developed to perform grid-based algorithms for cluster detection and case-control spatial epidemiology. We will present the principles of the software, its conditions of use, and will describe in some detail its application to the search of candidate environmental factors of Type 1 Diabetes.

## Methods

### Description of FGBASE

FGASE was devised to meet the needs of environmental epidemiologists in the two directions above mentioned:

- "Data driven" where the epidemiologist searches if there are clusters of cases which can later be interpreted. In this case, the data consists of the positions of the patients and the positions of the controls (or the density of the underlying at risk population).
- "Hypothesis driven" where the epidemiologist wants to test whether cases of a disease of interest are differently exposed than controls to environmental factors which are geographically specified. In this case the data are the positions of the patients and of the controls on the map, and the positions of the environmental points of interest.

The principle of the method is to use a high-resolution grid to avoid the computer costly computations of distances, as outlined in the introduction.

#### Representation of geographical information in FGBASE

**Choosing a geographical grid standard** Although the software will run with any type of grid, the use of equal area grids based on the Lambert Azimuthal Equal Area (LAEA) projection system is encouraged. This type of grid is recommended by the Directive 2007/2/E, which was adopted by the European Parliament in March 2007. This directive aims at establish an Infrastructure for Spatial Information in the European community (INSPIRE) for environmental policies [14]. The INSPIRE directive recommends the use of the Lambert Azimuthal Equal Area (ETRS89-LAEA) projection for pan-European spatial analysis and reporting when true area representation is required [15]. For each European country, population grids of this type can be downloaded from the European Commission’s Eurostat website [16].

**Figure 2:**
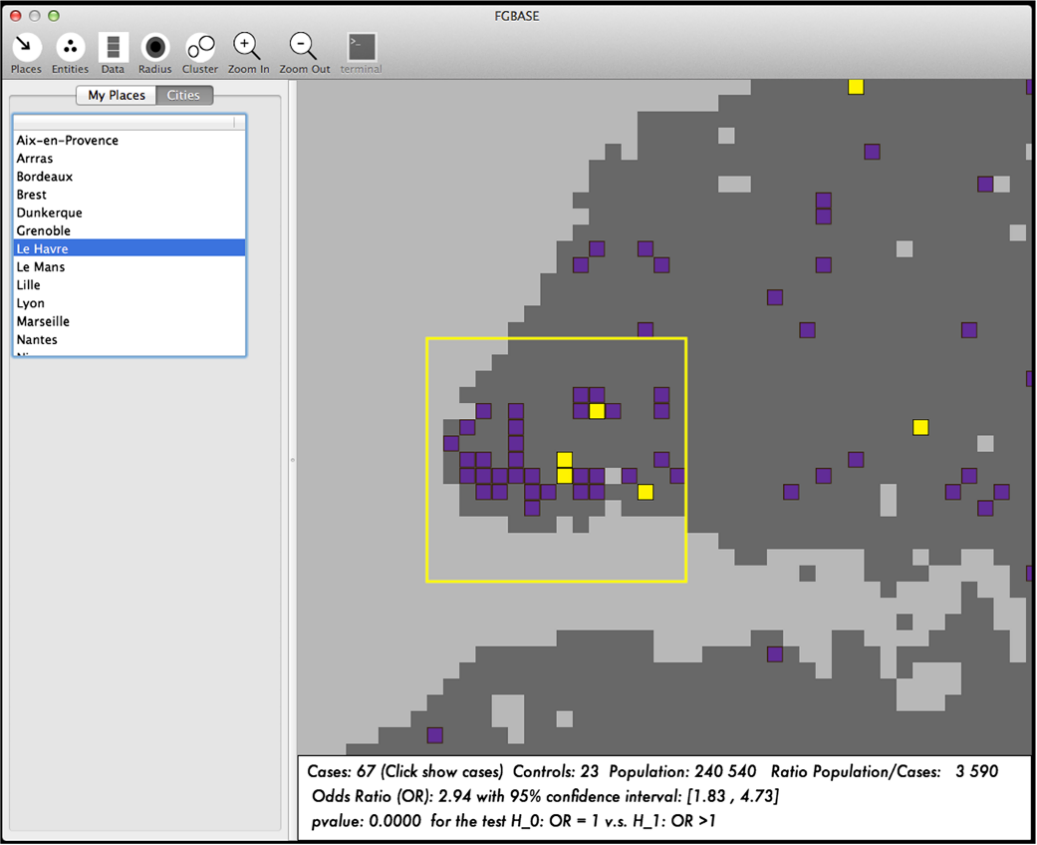
**Interactive map of the software FGBASE.** The interactive map (centered here on the city of Le Havre, France) displays the cells of the grid. These square cells are color coded according to user defined criteria. Here the following color codes are used: light grey if the square is uninhabited, dark grey if the square is inhabited, purple if the square contains cases of the disease, yellow if the square contains 2 or more cases *and* the population to case ratio of the square is below 1500. The map has a yellow selection rectangle, which the user can move and resize. The panel below the map displays statistics calculated for the region enclosed in the selection rectangle. The user can load a list of cities with their geographical cordinates, for easy access through the left panel.

**Projecting the data onto the geographical grid** Epidemiological data generally involves geographical coordinates encoded in the WGS84 coordinate system. WGS84 is the World Geodetic System established in 1984, which is well known through its use in the Global Positioning System (GPS). To project WGS84 coordinates to a grid based on the Lambert Azimuthal Equal Area (LAEA) projection system, the first step is to convert WGS84 coordinates to LAEA coordinates. This coordinate transformation can be done with the GDAL (Geospatial Data Abstraction Library) see [17]. One needs to know the EPSG codes of the source and target coordinate systems, which are (EPSG: 4326) for WGS84 and (EPSG: 3035) for ETRS89-LAEA. We provide a python script with which users can project their WGS84 coordinate data to ETRS89-LAEA grid coordinates for use in FGBASE software. The software FGBASE displays the geographical grid as an interactive map (see Figure 2).

**Representing the points of interest in the hypothesis driven approach** The grid-based approach is well suited for examining the effect of specific environmental factors, when these environmental factors can be located to specific points on the map. These can be isolated points (such as factories or waste dumps). More generally, curves and surfaces can be modeled as collections of points though sampling. This enables the grid-based approach to be applied to the study of the possible environmental hazards associated with a highway, a river, or specific agricultural cover using pesticides.

### Using FGBASE to test specific environmental factors in the hypothesis-driven approach

Three data sets are necessary: (i) locations of cases (ii) locations of controls (iii) locations of the environmental points of interest (e.g. pollution source points). These spatial entities can be labeled with an attribute, that we call "class" in order to group all those which share a same characteristic that the user will specify, based on his domain specific knowledge. For example (see the case study, below), the "entities" are factories that emit pollutants. A "class" is a category of polluting chemical (e.g. hydrazine). All "entities" (factories) that emit hydrazine belong to the same "class". This is being used by the software when comparing the distances of cases and controls to the entities (factories) that are of the same class (emitting hydrazine).

**Figure 3:**
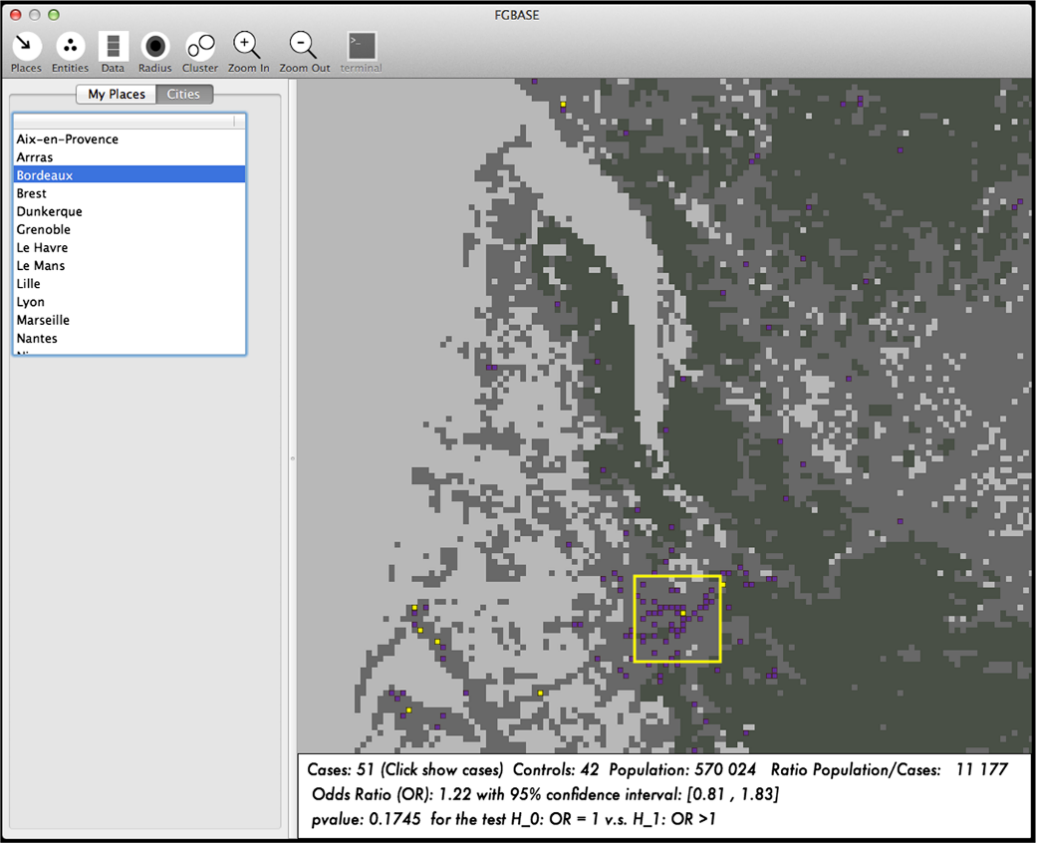
**Loading environmental factors into FGBASE.** Loading vineyards from the CLC land use database into the FGBASE software. Here the map is centered at Bordeaux, France. Vineyards account for a substantial portion of pesticide use. Analysis performed with the software does not show a link between this environmental factor and type 1 diabetes.

**Figure 4:**
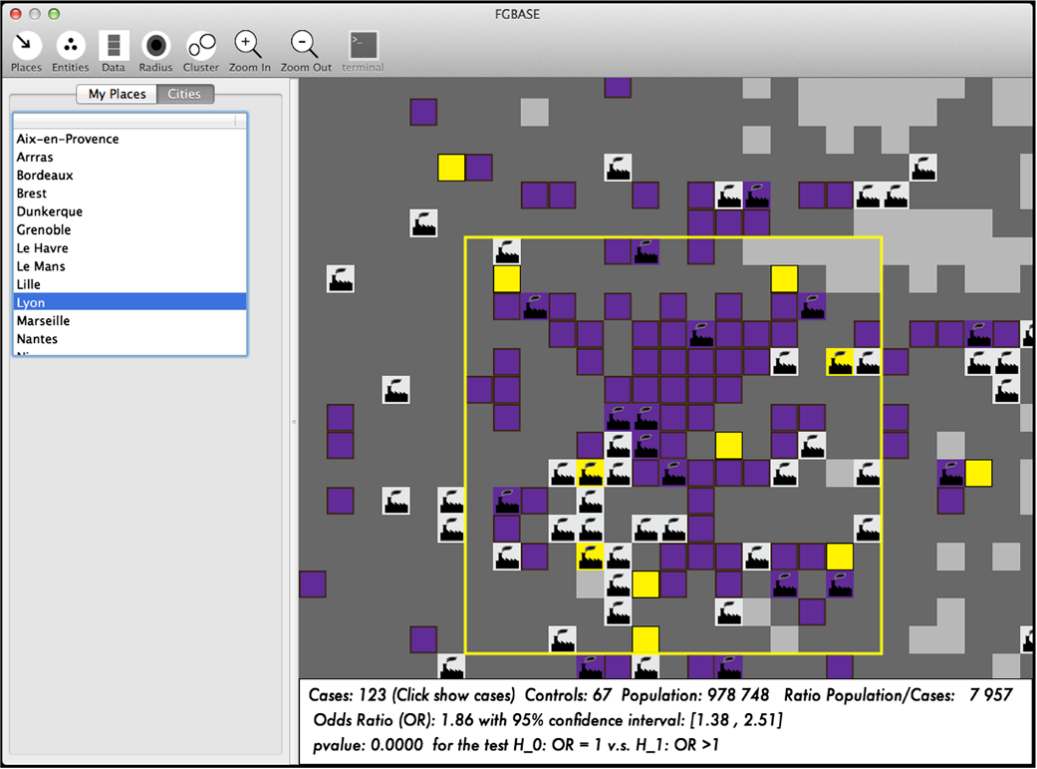
**Loading into FGBASE a data set of spatial entities grouped into classes: the IREP factories grouped by chemical emissions.** The spatial entities (IREP factories) are loaded in the FGBASE software and displayed on the interactive map. These entities are grouped into classes based on the chemicals which they have emitted (an entity can be assigned to several classes). The hypothesis driven algorithm tests each class of entities, one after the other, by reading from the grid the number of cases and controls at several different levels of proximity from entities in that class.

For each environmental under study, three levels of exposure are defined: (H = high, M = medium or L = low exposure). The distance to the point of interest determines these exposure levels based on user-defined thresholds. Individuals located in cells which are close to an entity in the class, are classified as highly exposed. Individuals located in cells which are a bit farther from an entity in the class, are classified as having medium level of exposure. Individuals located in cells, which are yet farther from an entity in the class, are classified as having a low level of exposure. Finally, individuals even farther out are classified as not exposed. Then the software computes a case control 2x2 contingency table for each level of exposition (H, M and L) versus the exposition 0, finally, the software computes the odds ratio and their 95% confidence intervals, and a test for tendency to check if there is a dose-response relationship. If (OR/H) > (OR/M) > (OR/L) significantly, then there is indication of a dose dependent association. The environmental factors are displayed on the interactive map of the software FGBASE (see Figure 3 and Figure 4).

### Using FGBASE to find cluster of cases using the data-driven option

In this option, the locations of cases are examined in the context of either a set of control locations or an underlying population density. A cluster search is performed to determine areas where the number of cases is much larger than would be expected by chance. Two datasets are necessary: (i) locations of cases (ii) locations of controls or appropriate population density data. For example, for all European countries, population grids can be downloaded from the European Commission’s Eurostat website [16]. (For non-European countries a solution could be to use the LandScan™ population density distribution see [18], but the user must ensure his/her cases and controls are projected to the same grid as the one used by LandScan™).

## Results

### A case study with FGBASE: patients with type 1 diabetes (T1D)

The development of FGBASE was driven by a program of search of possible environmental factors of Type 1 Diabetes (T1D). Indeed, in several European countries, the incidence of T1D continues to progress rapidly and has doubled since the 80′s in children aged less than 5 years [19]. The reason for this cannot be genetic as the observed variations occurred during a short time when the genetic structure of the population did not change. Numerous case-control studies of environmental associations with T1D have examined specific candidate factor approaches ( [20–23]) but no single factor has gained further credit in the causation of T1D ( [24, 25]).

The population used in this example is made with the participants of "Isis-Diab", an ongoing prospective cohort of T1D patients recruited since 2007 by the Isis-Diab Network composed of 99 diabetic centers covering almost all French regions (see description and list of participating centers in Additional file 1: Table S1). The main objective of the Isis-Diab program is the exploration of environmental and gene-environment interaction in patients with T1D. Inclusion criteria for the current study were T1D occurring in children aged less than 15 years. T1D was defined according to the American Diabetes Association [26], and by positive autoantibodies to GAD, insulin, and/or IA2. All studied patients were born in France. Data consist of clinical, genomic, and environmental exposures. Here, in the example shown to exemplify the "hypothesis" driven option of FGBASE, we do not consider the clinical and genetic characteristics of the patients and we focus on a single source of pollution described in the next section.

#### The environmental exposures analyzed

In France, all industrial polluting industries are registered and must provide data on the polluting emissions they are responsible of. Data are in the IREP database (see [27]) that contains comprehensive data (=93 809) on polluting emissions regarding 159 registered chemicals. The data set consists of three database tables. The first table contains a list of industrial entities such as factories. (total: n = 12 173) Each entity comes with a numerical identifier (entityID), as well as its geographical coordinates. The second table provides a list of 160 chemicals, each one having a numerical identifier (chemID). The third table contains a comprehensive list of chemical emissions over a period of 10 years (from 2000 to 2009). Each emission is provided with its date, its entityID and its chemID. This data set naturally fits into the framework of spatial entities grouped into classes. Here the classes are the registered chemicals (m = 160), and each class is associated to the entities that have emitted that chemical. The entities are mapped to the grid using their geographical coordinates.

"Hypothesis driven" option: In the hypothesis driven option cases must be compared to controls. Here we have defined "virtual controls" that are randomly taken over France in places of comparable density to those of cases. Strength of this method is that the definition of controls, and the sampling issues, can be addressed by replicating the algorithm as many times that needed. In the example, we chose to take 100 series of 4507 virtual controls, each series being compared to the cases. The algorithm will test each class separately, one after the other. This ensures stronger statistical power but is submitted to the classical false discovery issues.

Using the data-driven option, zones most likely to be clusters of T1D cases where discovered in the vicinity of the cities of Le Havre, Dunkerque, Toulouse and Nantes (see Table 1).

**Table 1:** **Candidate clusters of T1D cases discovered by the FGBASE using cases from the Isis-Diab cohort**

Cluster candidate	Cluster candidate 1	Cluster candidate 2	Cluster candidate 3	Cluster candidate 4
Location	Next to Le Havre	Next to Dunkerque	Next to Toulouse	Next to Nantes
LAEA -ETRS89 grid coordinates of the rectangular zone:	[(3610,2979), (3610,2981), (3608,2981), (3608,2979)]	[(3774,3126), (3774,3130), (3783,3130), (3783,3126)]	[(3627,2313), (3627,2315), (3631,2315), (3631,2313)]	[(3446,2746), (3446,2750), (3453,2750), (3453,2746)]
WGS84 coordinates of rectangular zone:	[(0.15368,49.4943), (0.150047,49.5121), (0.122639,49.5097), (0.126282,49.4919)]	[(2.19376,50.9823), (2.18771,51.018), (2.31538,51.0265), (2.32132,50.9908)]	[(1.40999,43.5677), (1.40733,43.5856), (1.45649,43.5897), (1.45914,43.5718)]	[(1.59501,47.2094), (-1.60299,47.2449), (-1.51148,47.2548), (-1.5035,47.2192)]
Number of cases	8 cases	13 cases	13 cases	41 cases
Number of controls	3 controls	0 controls	4 controls	16 controls
log of the numerator of Kulldorff’s likelihood ratio:	-6240.36	-6235.35	-6238.47	-6235.09

Using the data-driven option of the FGBASE software, zones most likely to be clusters of T1D cases where discovered in the vicinity of the cities of Le Havre, Dunkerque, Toulouse and Nantes.

### Results with the hypothesis driven option

Using the hypothesis driven option and correcting for multiple comparisons, the polluting chemicals in the IREP dataset did not show a statistically significant association with T1D.

## Discussion

### Comparisons with the SaTScan™ software

SaTScan™ [12] is a software program which implements the spatial scan statistic. It is very widely used (the SaTScan™ user guide [28] list over a hundred public health papers which have used it to obtain results in a wide range of studies). Despite its quality and wide user base, SaTScan™ has a certain number of drawbacks which warrant the existence of alternative software tools such as FGBASE. The first drawback is that while SaTScan™ is freely downloadable, it is not open source. This strongly limits its customizability, as the users cannot add and modify features to fit their needs. For example, we (the authors) have benefited from being able to customize the source code of the open source genomic analysis tool Plink [29] to add certain statistical tests which we needed but were not implemented in the original software. Another advantage of open source software is that after a while, user scrutiny of the source code reduces the number of bugs and security flaws in the software [30]. The benefits of open source software in the area of geographical information systems has been studied in [31].

A second drawback of SaTScan™ is that it uses circles. From the SaTScan™ user guide [28]: "With latitude/longitude coordinates, what planar projection is used? No projection is used. SaTScan™ draws perfect circles on the spherical surface of the earth". As discussed in this paper, the use of circles and distance calculations, is computationally much slower than using grid projections. Increasing the speed of SaTScan™ through the use of cloud services has been proposed in [13], it is clear however that using faster algorithms is a preferable (and less expensive) solution.

A third limitation of SaTScan™ is the lack of cartographic output [32], while this can be addressed though the use of an external macro [32], an integrated map as the one available in FGBASE, increases the ease of use when viewing clusters. Finally a fourth limitation of SaTScan™, is that it addresses only cluster searches and not the testing of environmental factors. By combining both types of analyses (cluster searches and testing of environmental factors) in a single software, FGBASE facilitates the interpretation of clusters in terms of environmental factors (both clusters and environmental factors are displayed on the same map).

### Types of environmental factors handled by FGBASE

The "hypothesis driven" grid-based algorithm described in this paper and implemented in the companion software (FGBASE) is well suited in the case of environmental factors, which can be located at specific points. By sampling curves and surfaces as collections of points, these are also well handled. The key aspect is that the environmental factors studied should be of a binary nature, in other words at any given location the factor should either be present or absent. This is the case for factories, power lines, highways and fields of specific crops. This is not the case for factors of a continuous nature such as temperature, atmospheric concentrations of a given gas or particle, or ultraviolet index.

## Conclusions

Grid-based algorithms for mining spatial epidemiological data provide advantages in terms of computational complexity and improve the speed of computations. This work starts by examining suitable geographical grids, and how epidemiological data is projected to such a grid. Based on this framework, data-mining algorithms are introduced which enable both a data-driven approach and a hypothesis-driven approach. These algorithms enable rapid discovery of clusters of cases as well the testing of specific environmental factors. A new open-source software tool (FGBASE) implementing these algorithms, is presented together with a case study of its use on the "Isis-Diab" cohort of French T1D cases. We hope that these methods and this software tool (FGBASE) will lower the computational barriers to entry for those performing epidemiological research.

FGBASE can be accessed at http://www.fgbase.org.

## Electronic supplementary material

Additional file 1: Table S1: List of the 95 diabetic centers participating to the Isis-Diab network by alphabetic order. The Isis-Diab study is coordinated by the INSERM unit 986. The principal investigators of the Isis-Diab study are Pierre Bougnères and Alain-Jacques Valleron (see http://www.isis-diab.org/ for an interactive map showing the geographical repartition of the centers, and the numbers of patients followed by each center). (DOCX 74 KB)

## References

[1] Kulldorff M (1997). A spatial scan statistic. Commun Stat-Theor Methods.

[2] Kulldorff M, Nagarwalla N (1995). Spatial disease clusters: detection and inference. Stat Med.

[3] Waller LA, Gotway CA (2004). Applied spatial statistics for public health data.

[4] Lawson AB, Denison DG (2010). Spatial cluster modelling.

[5] Lawson A, Biggeri A, Böhning D, Lesaffre E, Viel J-F, Bertollini R (1999). Disease mapping and risk assessment for public health.

[6] Assuncao R, Costa M, Tavares A, Ferreira S (2006). Fast detection of arbitrarily shaped disease clusters. Stat Med.

[7] Bithell J (1992). Statistical methods for analysing point-source exposures. Geographical and Environmental Epidemiology: Methods for Small Area Studies.

[8] Bithell JF, Stone RA (1989). On statistical methods for analysing the geographical distribution of cancer cases near nuclear installations. J Epidemiol Community Health.

[9] Diggle PJ (1990). A point process modelling approach to raised incidence of a rare phenomenon in the vicinity of a prespecified point. J R Stat Soc A Stat Soc.

[10] Stone RA (1988). Investigations of excess environmental risks around putative sources: statistical problems and a proposed test. Stat Med.

[11] Jacquez GM, Greiling DA (2003). International Journal of Health Geographics. Int J Health Geogr.

[12] Kulldorff M (2009). Information Management Services, Inc. SaTScanTM v8. 0: Software for the spatial and space-time scan statistics.

[13] Price RC, Pettey W, Freeman T, Keahey K, Leecaster M, Samore M, Tobias J, Facelli JC: **SaTScan on a Cloud: On-Demand Large Scale Spatial Analysis of Epidemics.** *Online J Public Health Inform* 2010.,**2**(1)**:** 10.5210/ojphi.v2i1.2910 PMC3615753 23569576

[14] Fleming DM, Schellevis FG, Falcao I, Alonso TV, Padilla ML (2001). The incidence of chickenpox in the community. Lessons for disease surveillance in sentinel practice networks. Eur J Epidemiol.

[15] Annoni A, Bernard L, Lillethun A, Ihde J, Gallego J (2004). Short Proceedings of the 1st European Workshop on Reference Grids.

[16] Epstein PR (2002). Climate change and infectious disease: stormy weather ahead?. Epidemiology.

[17] *GDAL - Geospatial Data Abstraction Library: Version 1.10.1*. Open Source Geospatial Foundation; 2014. [ http://www.gdal.org/]

[18] Dobson JE, Bright EA, Coleman PR, Durfee RC, Worley BA (2000). LandScan: a global population database for estimating populations at risk. Photogramm Eng Remote Sens.

[19] Patterson CC, Dahlquist GG, Gyürüs E, Green A, Soltész G (2009). Incidence trends for childhood type 1 diabetes in Europe during 1989–2003 and predicted new cases 2005–20: a multicentre prospective registration study. Lancet.

[20] Mohr S, Garland C, Gorham E, Garland F (2008). The association between ultraviolet B irradiance, vitamin D status and incidence rates of type 1 diabetes in 51 regions worldwide. Diabetologia.

[21] Knip M, Virtanen SM, Seppä K, Ilonen J, Savilahti E, Vaarala O, Reunanen A, Teramo K, Hämäläinen A-M, Paronen J (2010). Dietary intervention in infancy and later signs of beta-cell autoimmunity. N Engl J Med.

[22] Karlén J, Faresjö T, Ludvigsson J (2012). Could the social environment trigger the induction of diabetes related autoantibodies in young children?. Scand J Public Health.

[23] Hober D, Alidjinou EK (2013). Enteroviral pathogenesis of type 1 diabetes: queries and answers. Curr Opin Infect Dis.

[24] Forlenza GP, Rewers M (2011). The epidemic of type 1 diabetes: what is it telling us?. Curr Opin Endocrinol Diabetes Obesity.

[25] Nokoff N, Rewers M (2013). Pathogenesis of type 1 diabetes: lessons from natural history studies of high‒risk individuals. Ann N Y Acad Sci.

[26] Association AD (2009). Diagnosis and classification of diabetes mellitus. Diabetes Care.

[27] D’Agostino RBSR, Massaro JM, Sullivan LM (2003). Non-inferiority trials: design concepts and issues - the encounters of academic consultants in statistics. Stat Med.

[28] Kulldorff M (2006). SaTScanTM User Guide.

[29] Purcell S, Neale B, Todd-Brown K, Thomas L, Ferreira MA, Bender D, Maller J, Sklar P, De Bakker PI, Daly MJ (2007). PLINK: a tool set for whole-genome association and population-based linkage analyses. Am J Hum Genet.

[30] Hoepman J-H, Jacobs B (2007). Increased security through open source. Commun ACM.

[31] Steiniger S, Hay GJ (2009). Free and open source geographic information tools for landscape ecology. Ecol Inform.

[32] Abrams AM, Kleinman KP (2007). A SaTScan™ macro accessory for cartography (SMAC) package implemented with SAS® software. Int J Health Geogr.

