# Supplementary material for: An open source software for fast grid-based data-mining in spatial epidemiology (FGBASE)
Source: Int J Health Geogr. 2014 Oct 30;13:46. doi: 10.1186/1476-072X-13-46 (PMC4233060; doi:10.1186/1476-072X-13-46)
Supplement: Supplementary file 1 — Additional file 1: Table S1: List of the 95 diabetic centers participating to the Isis-Diab network by alphabetic order. The Isis-Diab study is coordinated by the INSERM unit 986. The principal investigators of the Isis-Diab study are Pierre Bougnères and Alain-Jacques Valleron (see http://www.isis-diab.org/ for an interactive map showing the geographical repartition of the centers, and the numbers of patients followed by each center). (DOCX 74 KB) [file 12942_2014_610_MOESM1_ESM.docx]

| **CENTER** | **PRINCIPAL INVESTIGATOR** |
| --- | --- |
| AIX EN PROVENCE | Dr Dominique THEVENIEAU |
| AMIENS (Diabétologie) | Dr Rachel DESAILLOUD |
| AMIENS (Pédiatrie) | Dr Helene BONY-TRIFUNOVIC |
| ANGERS (Diabétologie) | Dr Pierre-Henri DUCLUZEAU |
| ANGERS (Pédiatrie) | Pr Regis COUTANT |
| ANGOULEME | Dr Gerard POINTECOUTEAU |
| ARMENTIERES | Dr Sophie CAUDRELIER |
| ARRAS | Dr Armelle PAMBOU |
| AURILLAC | Dr Emmanuelle DUBOSCLARD |
| AVIGNON (Diabétologie) | Dr Eric BENAMO |
| AVIGNON (Pédiatrie) | Dr Florence JOUBERT |
| BAR LE DUC | Dr Philippe JAN |
| BELFORT-MONTBELIARD | Dr Estelle MARCOUX |
| BESANCON | Dr Anne-Marie BERTRAND |
| BETHUNE | Dr Chantal STUCKENS |
| BORDEAUX | Dr Pascal BARAT |
| BOULOGNE BILLANCOURT | Dr Chantal STHENEUR |
| BOULOGNE SUR MER | Dr Sylviane FOURNIER |
| BREST (Diabétologie) | Pr Veronique KERLAN |
| BREST (Pédiatrie) | Dr Chantal METZ |
| BRIVE | Dr Anne FARGEOT-ESPALIAT |
| CAEN | Pr Yves REZNIC |
| CAMBRAI | Dr Ramona NICOLESCU |
| CARCASSONNE | Dr Frédéric TRONC |
| CHALONS EN CHAMPAGNE | Dr Hervé GRULET |
| CHARTRES | Dr Arnaud MONIER |
| CHOLET | Dr Catherine RADET |
| CLAMART | Dr Vincent GAJDOS |
| CLERMONT FERRAND | Dr Daniel TERRAL |
| COMPIEGNE | Dr Christine VERVEL |
| DIJON | Dr Candace BEN SIGNOR |
| DOUAI | Dr Daniel DERVAUX |
| DUNKERQUE | Dr Guy-Andre LOEUILLE |
| EPINAL | Dr Françoise POPELARD |
| GAP | Dr Agnès GUILLOU |
| HYERES | Dr Jamil KHOURY |
| LA ROCHE SUR YON | Dr Jean-Pierre BROSSIER |
| LA ROCHELLE | Dr Didier GOUET |
| LAVAL | Dr Joachim BASSIL |
| LE HAVRE | Dr Bernard LE LUYER |
| LE MANS | Dr Françoise LABAY |
| LENS | Dr Isabelle GUEMAS |
| LILLE | Pr Jacques WEILL |
| LILLE (Clinique) | Dr Jean-Pierre CAPPOEN |
| LIMOGES (Diabétologie) | Dr Sylvie NADALON |
| LIMOGES (Pédiatrie) | Dr Anne LIENHARDT-ROUSSIE |
| LYON | Pr Marc NICOLINO |
| MARSEILLE (La Timone) | Pr Gilbert SIMONIN |
| MARSEILLE (St Joseph) | Dr Jacques COHEN |
| MARSEILLE CHU NORD | Dr Catherine ATLAN |
| MAUBEUGE | Dr Agnes TAMBOURA |
| MERIGNAC | Dr Herve DUBOURG |
| MONT-DE-MARSAN | Dr Marie-Laure PIGNOL |
| MONTBELIARD | Dr Estelle MARCOUX |
| MONTFERMEIL | Dr Philippe TALON |
| NANCY | Dr Stephanie JELLIMANN |
| NANTES (Diabétologie) | Dr Lucy CHAILLOUS |
| NANTES (Pédiatrie) | Dr Sabine BARON |
| NICE | Dr Marie-Noëlle BORTOLUZZI |
| NICE (Hôpital Lenval) | Dr Elisabeth BAECHLER |
| NIMES | Dr Randa SALET |
| NIORT | Dr Ariane ZELINSKY-GURUNG |
| PALAVAS LES FLOTS | Dr Fabienne DALLAVALE |
| PARIS BICETRE | Pr Pierre BOUGNERES |
| PARIS BICETRE | Pr Pierre BOUGNERES |
| PARIS HOTEL DIEU | Dr Etienne LARGER |
| PARIS LARIBOISIERE | Dr Marie LALOI-MICHELIN |
| PARIS SAINT ANTOINE | Pr Alain-Jacques Valleron |
| PARIS ST LOUIS | Dr Jean-François GAUTIER |
| PAU | Dr Benedicte GUERIN |
| PONTOISE | Dr Laetitia PANTALONE |
| REIMS | Dr Celine LUKAS |
| RENNES (Diabétologie) | Dr Isabelle GUILHEM |
| RENNES (Pédiatrie) | Dr Marc DE KERDANET |
| ROUEN | Dr Marie-Claire WIELICKZO |
| SAINT ETIENNE | Dr Odile RICHARD |
| SAINT MALO | Dr Anne JOSSENS |
| SAINT-AVOLD | Dr François KURTZ |
| SAINT-LO | Dr Norbert LAISNEY |
| SAINT-NAZAIRE | Dr Guilhem PARLIER |
| SAINTES | Dr Catherine BONIFACE |
| TARBES - Diabétologie | Dr Pierre Jean Louvet |
| TARBES - Pédiatrie | Dr Denis Dufillot |
| THIONVILLE | Dr Mirella CODREANU |
| TOULON | Dr Berthe RAZAFIMAHEFA |
| TOULOUSE | Dr Pierre GOURDY |
| TOULOUSE - Pédiatrie | Dr Pierre GOURDY |
| TOURS (Diabétologie) | Pr Pierre LECOMTE |
| TOURS (Pédiatrie) | Dr Myriam PEPIN-DONAT |
| TREMBLAY EN FRANCE | Dr Marie-Emmanuelle COMBES-MOUKHOVSKY |
| VALENCE | Dr Marina RAOULX |
| VALENCIENNES (Diabétologie) | Dr Odile VERIER-MINE |
| VALENCIENNES (Pédiatrie) | Dr Anne GOURDIN |
| VIENNE | Dr Catherine DUMONT |
| VILLEFRANCHE SUR SAONE | Dr Michele CHAMBON |
